# Misfolding and aggregation of nascent proteins: a novel mode of toxic cadmium action in vivo

**DOI:** 10.1007/s00294-017-0748-x

**Published:** 2017-09-21

**Authors:** Markus J. Tamás, Bruno Fauvet, Philipp Christen, Pierre Goloubinoff

**Affiliations:** 1 Department of Chemistry and Molecular Biology, University of Gothenburg, 405 30 Gothenburg, Sweden; 2 Department of Plant Molecular Biology, Lausanne University, 1015 Lausanne, Switzerland; 3 Department of Biochemistry, University of Zurich, 8057 Zurich, Switzerland

**Keywords:** Metal toxicity, Protein aggregation, Protein folding, *Saccharomyces cerevisiae*, Cadmium, Molecular chaperones

## Abstract

Cadmium is a highly poisonous metal and a human carcinogen, but the molecular mechanisms underlying its cellular toxicity are not fully understood. Recent findings in yeast cells indicate that cadmium exerts its deleterious effects by inducing widespread misfolding and aggregation of nascent proteins. Here, we discuss this novel mode of toxic heavy metal action and propose a mechanism by which molecular chaperones may reduce the damaging effects of heavy metal ions on protein structures.

## How cadmium acts at the level of organisms and cells

Human exposure to toxic metals and metalloids is increasing in many parts of the world due to geochemical contamination and industrial pollution. The heavy metal cadmium is considered genotoxic and neurotoxic, and exposure is associated with lung and kidney cancers. In addition, cadmium may be implicated in the progression of neurological disorders caused by protein misfolding (Chin-Chan et al. 2015; Maret and Moulis 2013; Tamás et al. 2014; Wang and Du 2013). At the cellular level, cadmium has been reported to affect redox homeostasis, interfere with the homeostasis of essential metals, cause DNA damage, impair DNA repair mechanisms, and perturb protein function and activity. At the molecular level, these toxic effects of cadmium have up to now been attributed mainly to its interactions with metalloproteins or other susceptible proteins (Bertin and Averbeck 2006; Beyersmann and Hartwig 2008; Maret and Moulis 2013; Tamás et al. 2014; Wang and Du 2013). Examples of cadmium binding to native, i.e., folded proteins, are abundant in the literature; however, most of these studies have used purified proteins and in vitro binding assays (Maret and Moulis 2013) and the importance of this mode of action for cellular toxicity has remained unclear. Recent findings in yeast cells indicate that cadmium exerts its deleterious in vivo effects by inducing widespread misfolding and aggregation of nascent proteins (Jacobson et al. 2017).

## Protein folding and quality-control

To perform their functions in the cell, most proteins must first fold into a defined three-dimensional structure, their native conformation. Misfolded proteins are not only inactive but also cytotoxic, as they may aggregate and/or interact inappropriately with other cellular components. Numerous pathological conditions are associated with protein misfolding and aggregation including several neurodegenerative diseases and age-related disorders, such as Alzheimer’s and Parkinson’s disease. To protect themselves against the harmful accumulation of protein aggregates, cells use an array of protein quality-control mechanisms. Molecular chaperones assist the folding of proteins into their functional conformation or help misfolded proteins to regain their native structures. Moreover, molecular chaperones are integral parts of protein degradation systems that clear cells from misfolded and aggregated protein conformers such as the proteasome, the autophagy pathway, and the lysosome/vacuole (Goloubinoff 2016; Hipp et al. 2014; Tyedmers et al. 2010).

## A novel mode of toxic metal action: cadmium induces misfolding and aggregation of nascent proteins in vivo

We have previously demonstrated that cadmium strongly inhibits the refolding of chemically denatured proteins in vitro at concentrations that affect native, i.e, completely folded, proteins only to a minor degree (Sharma et al. 2008). Prompted by this finding, we investigated whether cadmium would affect protein folding also in living cells. Using the yeast *Saccharomyces cerevisiae* as model system, we demonstrated that cadmium induces misfolding and aggregation of nascent proteins (Jacobson et al. 2017). In vivo and in vitro data furthermore indicated that cadmium-aggregated protein species may form seeds that, in a gain-of-function mechanism, increase the misfolding and aggregation of other sensitive proteins. In addition, we found that zinc protected nascent polypeptides from cadmium-induced aggregation, raising the possibility that cadmium may displace zinc in metalloproteins and form complexes with alternative ligands. Zinc not only prevented cadmium-induced protein aggregation but also improved the cadmium tolerance of yeast cells. Similarly, yeast mutants with reduced capacity to protect the proteome from cadmium-induced aggregation or to clear the cytosol from protein aggregates proved more sensitive to cadmium than wild-type cells (Jacobson et al. 2017). Together, these results suggest that misfolding and aggregation of nascent proteins represent an important component of cadmium toxicity.

Like cadmium, arsenite (As^3+^) (Ibstedt et al. 2014; Jacobson et al. 2012) and chromium (Cr^6+^) (Holland et al. 2007) also cause protein aggregation in yeast cells. Whilst arsenite and cadmium directly interfere with protein folding (Jacobson et al. 2012, 2017), chromium triggers protein aggregation by enhancing mRNA mistranslation. Mistranslation appears to be the primary cause of cellular toxicity by chromium (Holland et al. 2007). In contrast, neither cadmium (our unpublished data) nor arsenite (Jacobson et al. 2012) cause mRNA mistranslation to any major extent.

Cells possess a number of subcellular deposition sites where misfolded proteins may be stored or processed (Miller et al. 2015; Saarikangas and Barral 2016). Whether metal-induced protein aggregates are sequestered to such sites remains to be investigated.

## How do cadmium ions interfere with the folding of protein molecules?

Cadmium and other heavy metal ions can bind to the S, N, and O atoms of proteins, forming relatively weak monodentate as well as much more stable pluridentate complexes. The most important ligands of the metal ions are the thiol groups of cysteine residues, the imidazole groups of histidine residues, and the carboxylate groups of acidic amino acid residues. The dissociation constants of pluridentate complexes, as metalloproteins form them with their specific essential metal ions, approximately correspond to the product of the dissociation constants of the individual monodentate complexes of their ligands (Table 1). The complexes in metalloproteins show mostly a tetrahedral or octahedral geometry with four or six ligands, respectively (Gurd and Wilcox 1956; Kägi and Hapke 1984; Vallee and Ulmer 1972).

**Table 1:** Monodentate complexes of cadmium with functional groups of proteins: dissociation equilibrium constants and p*K*_a_ values

	*K*_d_′ at pH 7^a^	Approximate p*K*_a_ in proteins
Thiol group	2.5 µM	9.4
Imidazole group	2.0 mM	6.5
Carboxyl group	16 mM	4.6

^a^ *K*_d_′ is the apparent dissociation equilibrium constant at pH 7 of the reaction protein·Cd^2+^ ↔ protein + Cd^2+^ (Kägi and Hapke 1984). The *K*_d_′ values for Zn^2+^ are quite similar to those for Cd^2+^ (Gurd and Wilcox 1956; Krezel and Maret 2016)

During the process of folding, the protein backbone is flexible and the side chains with their potential metal ligands are not only more exposed to the solvent but also more flexible and motile; in any protein, these conditions favour the formation of stable pluridentate metal complexes with their highly specific geometry. Once the polypeptide chain has engaged in such a complex, it can no longer assume the native protein structure and will thus misfold and aggregate. Such metal-induced misfolding of proteins and the ensuing protein aggregation were observed both in vitro during the renaturation of chemically denatured proteins (Jacobson et al. 2012, 2017; Ramadan et al. 2009; Sharma et al. 2008) and in vivo during protein synthesis in yeast cells (Ibstedt et al. 2014; Jacobson et al. 2012, 2017). The in vitro refolding of denatured proteins and intracellular co-translational folding share, despite their obvious differences, a decisive feature; flexible, motile backbone segments and accessible side chains allow the formation of stable pluridentate metal complexes that cannot be accommodated in the final native structure. In contrast, in the native, completely folded protein molecule, part of the potential ligand groups are not accessible and, more importantly, the formation of pluridentate complexes with their specific geometry would require an extensive alteration of the native spatial structure which corresponds to the free-energy minimum of the polypeptide chain.

Which proteins are susceptible for metal-induced aggregation? Recent studies in yeast indicated that various stress conditions including the metalloid arsenite (Ibstedt et al. 2014; Weids et al. 2016), promoted the aggregation of similar types of proteins. These proteins appeared particularly susceptible for aggregation during translation/folding (Weids et al. 2016), suggesting that they never reached their native conformation. The above molecular mechanism by which heavy metal ions interfere with the intracellular folding of nascent polypeptide chains may safely be assumed to be operative with very many, if not the majority, of cellular proteins. Presumably, a multitude of parameters determine the degree of interference by metal ions with the folding of intracellular proteins. These parameters include among others; the type of metal ion and target protein, their intracellular concentrations, the concentrations of competing essential and xenobiotic metal ions, the amino acid sequence of the target protein, in particular content and mutual arrangement of potential ligand groups in side chains (thiol, imidazole, carboxylate) in the sequence and spatial structure, the folding pathway, rate of folding, structure of folding intermediates such as molten globule state, the concentration of competing essential and xenobiotic metal ions, and lastly the effect of cellular defence mechanisms against surplus metal ions (ion pumps, sequestration mechanisms) and against accumulation of misfolded/aggregated proteins (chaperones, sequestration, degradation).

Using the DnaK/DnaJ/GrpE chaperone system of *Escherichia coli*, we demonstrated that cadmium inhibits chaperone-assisted refolding of chemically denatured and heat-denatured proteins in vitro (Sharma et al. 2008). This observation raised the possibility that cadmium directly inhibits refolding chaperones. However, unlike previous findings with arsenite (Jacobson et al. 2012), cadmium did not interfere with resolution of protein aggregates formed by thermal stress (Jacobson et al. 2017), suggesting that in yeast, cadmium may not be a strong inhibitor of cytosolic chaperone action.

A different mechanism by which a xenobiotic metal may diminish the biological activity of a target metalloprotein is the substitution of the essential metal ion, e.g., zinc, by another heavy metal of the zinc group, e.g., cadmium, while the nascent polypeptide chain undergoes the folding process. In vitro metal replacement experiments with a number of metalloenzymes suggest that under these circumstances the protein would fold into its native structure provided that the substitute metal ion fits into the geometry of the complex. The nascent protein will accept the substitute metal ion, will not misfold and will not aggregate; its biological activity, however, might be substantially decreased (Kägi and Hapke 1984; Vallee and Ulmer 1972). We found that zinc strongly mitigates cadmium-induced protein aggregation in living yeast cells (Jacobson et al. 2017), indicating that cadmium–zinc exchange may occur in yeast during protein synthesis and folding.

## Unfolding chaperones may prevent heavy metal damages in proteins and avert prion-like aggregate propagation

A major function of molecular chaperones is to prevent the aggregation of nascent polypeptide chains or stress-unfolded proteins (the so-called holdases), assist the folding of proteins into their functional conformation (foldases), or help misfolded and aggregated proteins to regain their native structures (unfoldases) (Finka et al. 2016). Molecular chaperones have also been implicated in the formation and propagation of prions, i.e., misfolded proteins that have adopted an infectious amyloid conformation. In mammals, prion proteins are associated with several neurodegenerative diseases (Chernoff and Kiktev 2016; Lazarev et al. 2017). Molecular chaperones, which are often, albeit improperly (Finka et al. 2011) referred to as heat-shock proteins (HSPs), form about 5% of the total protein mass of unstressed eukaryotic cells, whilst the chaperone load may reach 10% of the total protein mass in cancer cells (Finka and Goloubinoff 2013). Like many other stress conditions, cadmium induces a substantial upregulation of chaperones in cells (Bertin and Averbeck 2006; Tamás et al. 2014). Half of the total chaperone mass is composed of members of the conserved Hsp90 and Hsp70 chaperone families that control all aspects of protein homeostasis in cells. Both act as nanomachines (Mattoo and Goloubinoff 2014) that use the energy of ATP hydrolysis to carry out numerous essential cellular processes, such as mediating the proper native folding of de novo synthetized or translocated polypeptides, promoting their proper assembly into native oligomers, and activating or inactivating them during various stages of cellular life. Importantly, by acting as polypeptide unfolding enzymes, some molecular chaperones can catalyse the conversion of toxic, stress- and/or mutation-induced protein aggregates, into natively refolded, harmless, biologically active proteins (Iosefson et al. 2012; Mattoo and Goloubinoff 2014; Priya et al. 2013; Sharma et al. 2009). Only when the repair of misfolded proteins has failed, may chaperones target the irreversibly damaged protein species to degradation by chaperone-gated proteases (Finka et al. 2016).

**Fig. 1:**
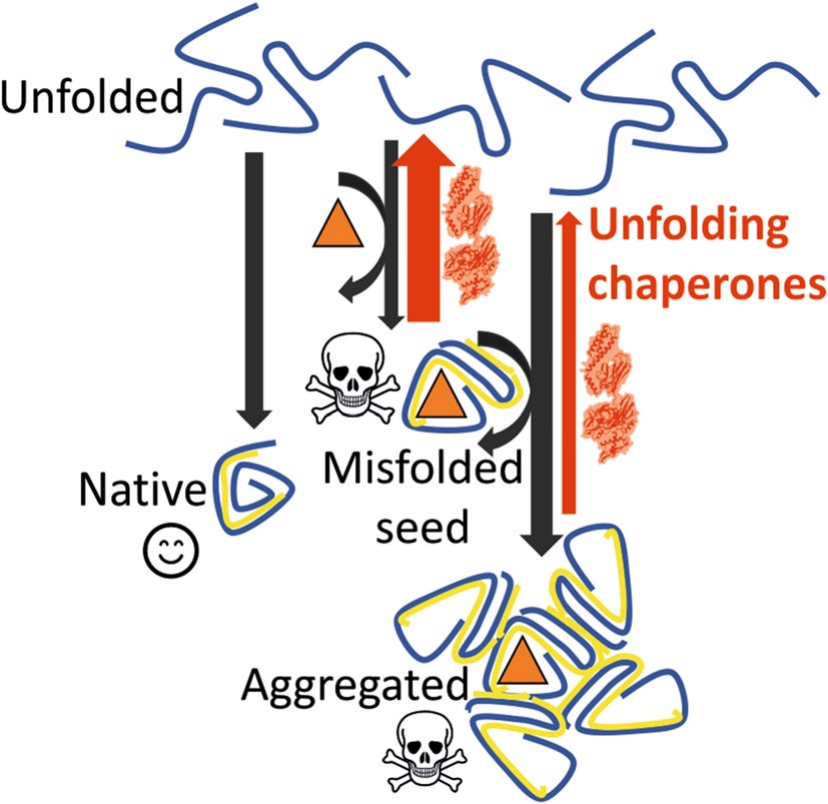
Mechanisms by which unfolding chaperones may reduce protein damage by heavy metal ions. A de novo synthetized or stress-unfolded polypeptide (top), can fold spontaneously into native, biologically active, harmless protein (left, smiley), or interact with a heavy metal ion (triangle) and misfold into a non-functional toxic species (middle, skull) that may further catalyse the misfolding of unfolded polypeptides, even when heavy metal ions are sub-stoichiometric, into more non-functional toxic species (right, skull). By their unfolding action, molecular chaperones (red arrows) may reduce the concentration of seeds that catalyse the formation of toxic protein aggregates

Although chaperones and other heat-shock proteins are induced by cadmium and other heavy metals or metalloids, it is not clear to what extent the rescuing of proteins contributes to tolerance (Tamás et al. 2014). How could an increased cellular concentration of chaperones counteract heavy metal toxicity? Chaperones such as Hsp70 and Hsp104 can act as catalytic polypeptide unfoldases capable of using the energy of ATP-hydrolysis to actively solubilize and reactivate misfolded protein aggregates (Finka et al. 2016). It is thus plausible that by converting metal-ion-induced misfolded and aggregated protein species into transiently active native proteins (Fig. 1; middle and right paths), chaperones might contribute to an increased cellular tolerance to heavy metals. Yet, because metal ions may affect both the native refolding pathway of chaperone-repaired (unfolded) aggregated proteins and also directly inactivate the chaperone machineries, elevated cellular metal concentrations would soon overwhelm and neutralize the protein quality-control machineries, leading to cell death (Goloubinoff 2016).

We have previously shown in vitro that during mild heat-denaturation, trace amounts of an aggregation-prone protein acted as proteinaceous infectious seeds that could dramatically accelerate the misfolding of another less thermolabile protein in excess. Remarkably, the presence of an active unfolding/disaggregating chaperone system, that effectively neutralized the seed formation, could optimally prevent seed-induced aggregation of the less labile abundant protein species (Ben-Zvi and Goloubinoff 2002). A similar seed neutralization effect by human Hsp70–Hsp110 chaperones was also observed with α-synuclein seeds causing protofibril formation in vitro, as in Parkinson’s disease (Finka et al. 2016). These observations raise the possibility that even low sub-toxic levels of heavy metals or metalloids in cells might trigger prion-like infectious propagations of toxic aggregates. Such aggregates might remain inconsequential in young tissues in which disaggregating and unfolding chaperones maintain the cellular concentration of seeds below the threshold for effective prion-like propagation. In aging cells, however, in which cellular levels of HSP chaperones are known to decrease (Ben-Zvi et al. 2009; Goloubinoff 2016; Shemesh et al. 2017), low levels of heavy metals might suffice to cause cell death. It is tempting to speculate that cells in aging tissues that fail to accumulate enough chaperones will also fail to counteract the formation of aggregation seeds (Fig. 1; right path), implying that even low concentrations of heavy metal ions taken in youth, might become toxic with age and initiate degenerative diseases associated with protein misfolding.

## Perspectives

Our recent studies in yeast cells have demonstrated that cadmium (Jacobson et al. 2017) and arsenite (Jacobson et al. 2012) severely interfere with the folding of nascent proteins and thus reduce cellular viability. As indicated by in vitro experiments (Sharma et al. 2008), heavy metals and metalloids other than cadmium and arsenite also perturb protein folding and likely manifest their toxicity through similar mechanisms. The clinical symptoms of acute and chronic metal poisoning are often vague with few metal-specific features. This rarity of metal-specific manifestations might reflect the interference of metal ions with the folding of a multitude of proteins as well as the consequences of the inactivation of chaperones and other repair systems that will again affect a multitude of misfolded proteins. To better appreciate the toxic and pathogenic effects of poisonous metals, detailed molecular understanding is required regarding the mechanisms by which they interfere with protein folding and promote protein aggregation in vivo, how such aggregates impair cellular functions, and how cells regulate the protein quality-control systems to protect against aggregate toxicity. Such knowledge may be expected to contribute to the development of new strategies for both disease prevention and treatment of metal poisoning.
